# Automated lung cancer assessment on 18F-PET/CT using Retina U-Net and anatomical region segmentation

**DOI:** 10.1007/s00330-022-09332-y

**Published:** 2023-01-10

**Authors:** T. Weikert, P. F. Jaeger, S. Yang, M. Baumgartner, H. C. Breit, D. J. Winkel, G. Sommer, B. Stieltjes, W. Thaiss, J. Bremerich, K. H. Maier-Hein, A. W. Sauter

**Affiliations:** 1 Department of Radiology, University Hospital Basel, University of Basel, Petersgraben 4, 4031 Basel, Switzerland; 2 Division of Medical Image Computing, German Cancer Research Center, Im Neuenheimer Feld 223, 69120 Heidelberg, Germany; 3 Department of Radiation Oncology, Pattern Analysis and Learning Group, Heidelberg University Hospital, Im Neuenheimer Feld 400, 69120 Heidelberg, Germany; 4 Department of Nuclear Medicine, University Hospital Ulm, Albert-Einstein-Allee 23, 89081 Ulm, Germany; 5 Institute of Radiology and Nuclear Medicine, Hirslanden Klinik St. Anna, St. Anna-Strasse 32, 6006 Lucerne, Switzerland

**Keywords:** Lung neoplasm, PET/CT, Neoplasm staging, Artificial intelligence, Deep learning

## Abstract

**Objectives:**

To develop and test a Retina U-Net algorithm for the detection of primary lung tumors and associated metastases of all stages on FDG-PET/CT.

**Methods:**

A data set consisting of 364 FDG-PET/CTs of patients with histologically confirmed lung cancer was used for algorithm development and internal testing. The data set comprised tumors of all stages. All lung tumors (T), lymphatic metastases (N), and distant metastases (M) were manually segmented as 3D volumes using whole-body PET/CT series. The data set was split into a training (*n* = 216), validation (*n* = 74), and internal test data set (*n* = 74). Detection performance for all lesion types at multiple classifier thresholds was evaluated and false-positive-findings-per-case (FP/c) calculated. Next, detected lesions were assigned to categories T, N, or M using an automated anatomical region segmentation. Furthermore, reasons for FPs were visually assessed and analyzed. Finally, performance was tested on 20 PET/CTs from another institution.

**Results:**

Sensitivity for T lesions was 86.2% (95% CI: 77.2–92.7) at a FP/c of 2.0 on the internal test set. The anatomical correlate to most FPs was the physiological activity of bone marrow (16.8%). TNM categorization based on the anatomical region approach was correct in 94.3% of lesions. Performance on the external test set confirmed the good performance of the algorithm (overall detection rate = 88.8% (95% CI: 82.5–93.5%) and FP/c = 2.7).

**Conclusions:**

Retina U-Nets are a valuable tool for tumor detection tasks on PET/CT and can form the backbone of reading assistance tools in this field. FPs have anatomical correlates that can lead the way to further algorithm improvements. The code is publicly available.

**Key Points:**

*• Detection of malignant lesions in PET/CT with Retina U-Net is feasible.*

*• All false-positive findings had anatomical correlates, physiological bone marrow activity being the most prevalent.*

*• Retina U-Nets can build the backbone for tools assisting imaging professionals in lung tumor staging.*

**Supplementary Information:**

The online version contains supplementary material available at 10.1007/s00330-022-09332-y.

## Introduction

It is well recognized that the positron emission tomography (PET) component of PET/CT with 18F-fluorodeoxyglucose (FDG) as radiotracer bears important metabolic information for tumor characterization and staging of lung cancer [1, 2]. Consequently, FDG-PET/CT became the standard for the diagnostic workup of patients with lung cancer [3]. But despite advancing technologies, imaging-based staging is still a challenging task [4] and both underdiagnosis and overdiagnosis are common problems [5]. Appropriate treatment is based on correct staging [6]. Due to the fast progression of the disease [7], initial mis-staging has a particularly negative impact on patients’ quality of life.

Automated lung tumor detection using artificial intelligence (AI) algorithms has shown promising results on high-resolution CT [8, 9]. However, particularly detection of advanced tumors on the CT component of PET/CTs with a slice thickness of 3 mm and acquisition in free-breathing technique is challenging for algorithms and currently not accurate enough [10]. Given this and the hybrid nature of PET/CT, it is advisable to use both PET and CT information during development of AI algorithms. So far, only few researchers have done so, and all focused solely on lung lesions (T) or lung nodules [11–14]. Further developments towards 3D detection of malignant lesions of different kinds (tumor, lymph node metastases, distant metastases) within the complete scan volume are needed to result in a clinically relevant, comprehensive solution assisting radiologists in TNM staging.

Recently, P. Jaeger and colleagues presented an application based on Retina U-Net for end-to-end object detection on medical data. It showed good results for the detection of breast cancer on MRI in both 2D and 3D [15]. Performance was superior to other approaches such as Mask R-CNN. For the first time, this approach is adapted, trained, and tested on a clinical data set of 384 PET/CTs of patients with histologically confirmed lung cancer of all stages. Furthermore, the algorithm is combined with an anatomical region segmentation to classify detected lesions into T, N, and M categories. We hypothesize that Retina U-Net is an effective tool for detection of T, N, and M lesions on PET/CT.

## Material and methods

Written informed consent for this retrospective study was waived by the regional ethics committee (Ethikkommission Nordwest- und Zentralschweiz).

### Case selection: internal data sets

**Fig. 1:**
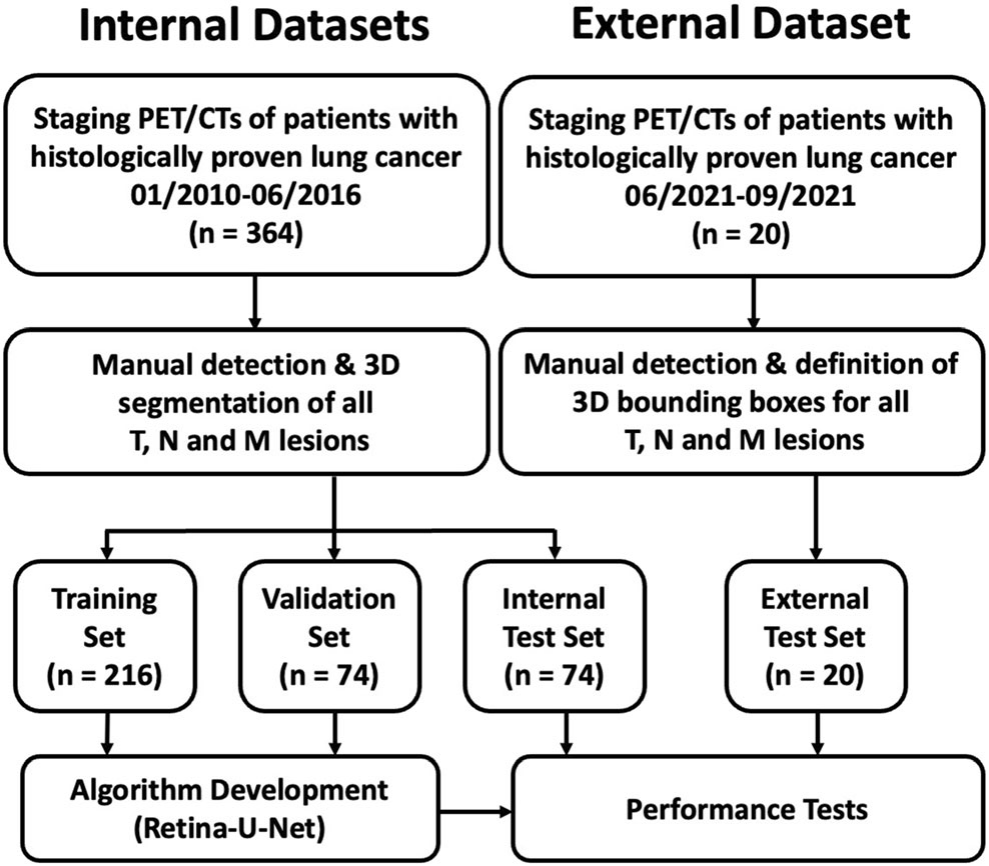
Study workflow

We identified FDG-PET/CTs of patients with histologically proven primary lung cancer acquired at our institution between 01/2010 and 12/2016. Selection criteria were protocol name, time period, and verified tumor histology according to the pathology archive. This resulted in 364 FDG-PET/CTs. Figure 1 shows the study workflow.

### Imaging protocols and reporting

PET/CT scans were performed on two integrated PET/CT systems: a Discovery STE with 16-slice CT (GE Healthcare) and a Biograph mCT-X RT Pro Edition with 128-slice CT (Siemens Healthineers). Scans were obtained 1 h after intravenous injection of 5 MBq FDG/kg body weight at glycemic levels below 10 mmol/L and previous fasting for at least 6 h. Technical details are provided in Supplement A. The clinical PET/CT reports had been created by residents in nuclear medicine and reviewed and signed by a board-certified radiologist and nuclear medicine physician in consensus.

### TNM annotation and image segmentation

After de-identification, the PET/CT of each patient was opened in a locally installed segmentation software (3D-Slicer, version 4.6.2). The TNM classification differentiates four main T categories (T1–T4; depending on size and features such as invasiveness), three N categories (N1–N3, depending on location), and one M-category [16]. As shown in Table 1, this study used the official TNM classification, slightly simplified by leaving out the sub-subcategories (e.g., T1a, T1b). Annotation and 3D image segmentation with reference to the anonymized written PET/CT report were performed by a dual-board-certified radiologist and nuclear medicine physician highly experienced in PET/CT reading (A.W.S., reader 1 [R1], 11 years of experience in cardiothoracic imaging, *n* = 141) as well as a supervised radiology resident with 4 years of professional experience (T.W., reader 2 [R2], *n* = 223). All of R2’s segmentation masks were visually checked and approved by R1. Each lesion was segmented as 3D volume defined by 2D regions of interest drawn on the contiguous transverse sections of the CT component. Fusion with PET information was available as additional information. A total of 60 lesions (20 T, 20 N, 20 M) were read independently by both readers and IoU calculated.

**Table 1:** Simplified TNM classification for lung cancer (8th edition) used in this study. Features in *italic* print

Category	Definition
T (primary tumor)	
T1	Tumor ≤ 3cm, *no features*
T2	Tumor > 3 but ≤ 5 cm; *or tumor involving visceral pleura/main bronchus, atelectasis*
T3	Tumor > 5 but ≤ 7 cm; *or invading chest wall/pericardium/phrenic nerve; or nodule in same lobe*
T4	Tumor > 7 cm; *or invading mediastinum, diaphragm, heart, great vessels, recurrent laryngeal nerve, carina, trachea, esophagus, spine; or tumor nodule in a different ipsilateral lobe*
N (lymph nodes)	
N1	Metastasis in ipsilateral pulmonary or hilar nodes
N2	Metastasis in ipsilateral mediastinal/subcarinal nodes
N3	Metastasis in contralateral mediastinal/hilar, or supraclavicular nodes
M (distant metastasis)	
M1	Metastasis in ipsilateral mediastinal/subcarinal nodes

### General algorithm characteristics: Retina U-Net

**Fig. 2:**
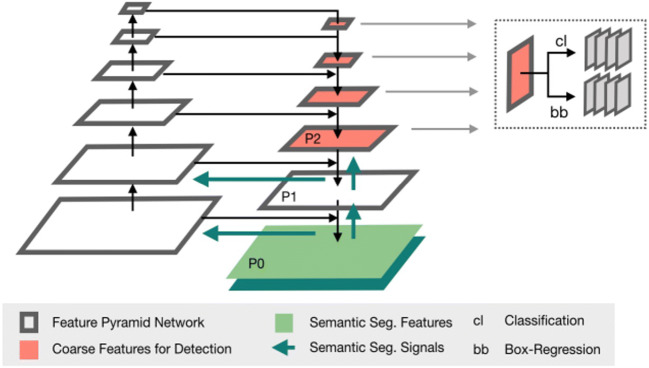
Retina U-Net architecture. The encoder-decoder structure resembles a U-Net. This segmentation model is complemented by a detection network for classification (cl) and bounding box regression (bb) operating on the lower (coarser) levels of the architecture so as to exploit object level features. The green arrows indicate the high quality training signals being backpropagated from an auxiliary segmentation task. From [15]

**Fig. 3:**
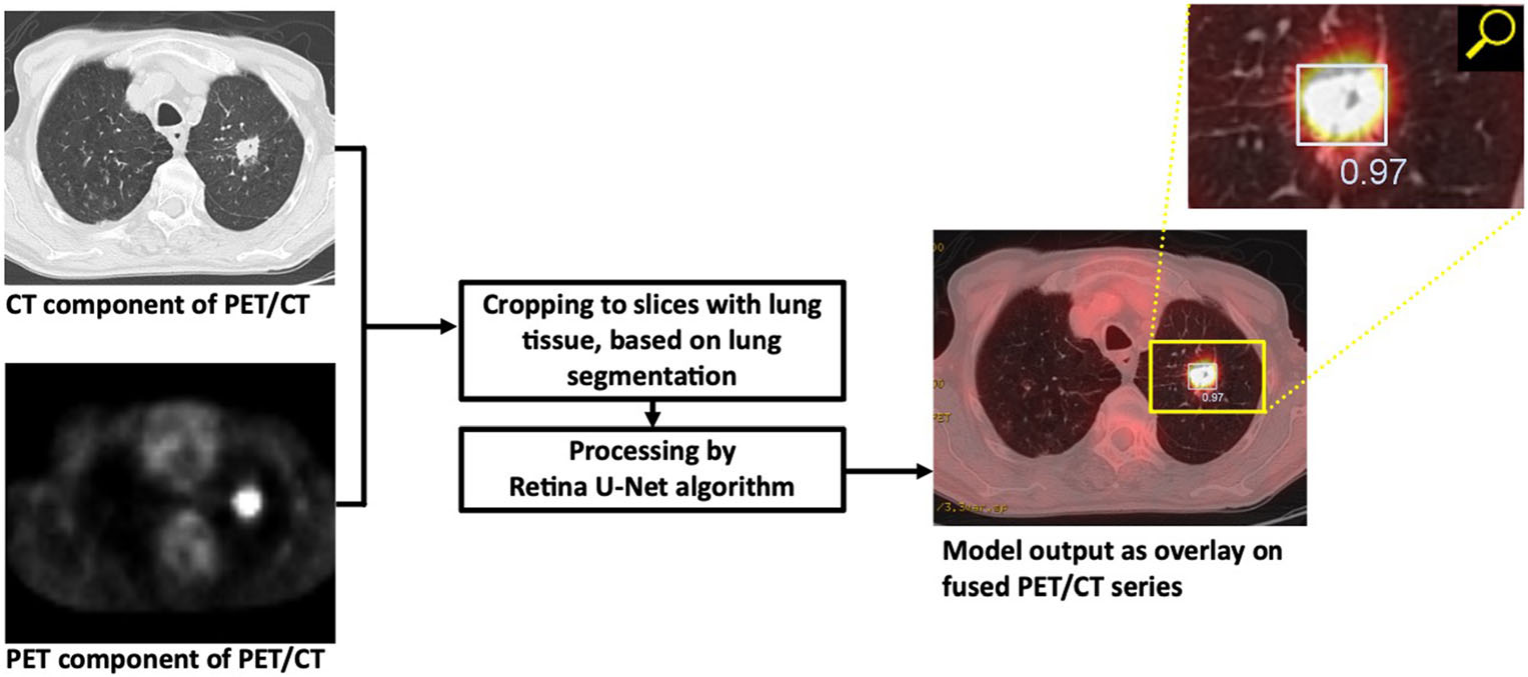
Schematic data processing workflow. Both the CT and PET component of the PET/CT serve as input for the Retina U-Net models, after cropping to the scan slices showing the lung. The model output are bounding boxes, in this example in light-blue color (top-right image) and indicating an adenocarcinoma (T1). These are compared to ground-truth bounding boxes generated from the 3D segmentations via IoU (intersection-over-union). Additionally, a classifier threshold is attached to each predicted bounding box (range 0.0–1.0) that quantifies the confidence of the Retina U-Net that a predicted lesion is a true lesion

Retina U-Net is a state-of-the art approach in medical object detection [15]. It recaptures training signals pixel-wise from segmentation supervision. The architecture used in this study is characterized by additional branches in the lower decoder levels for end-to-end object classification and bounding box regression (Fig. 2). Figure 3 shows the schematic data processing workflow. For the first time, the algorithm has been adapted and trained to detect T, N, and M lesions on PET/CTs.

### Training, validation, and testing

The complete internal data set was randomly split into a training (60%), validation (20%), and internal test set (20%). Intensity values were clipped at [−600, 1200], rescaled to [0, 1], and *z*-score normalized. Images were cropped on the *z*-axis to slices containing lung tissue according to a lung segmentation based on intensity-thresholding and connected component analysis. As the task of the algorithm is object detection, bounding boxes were created based on the manual 3D segmentations for training. Two other approaches including only T or only T&N lesions were also assessed, but performed worse and were not pursued further (see Supplement B). Intersection-over-union threshold was set to 0.1. Due to limitations in graphics processing unit (GPU) memory, 3D images were processed patch-wise. During training, patches containing foreground objects were oversampled to ensure the balance between foreground and background patches. Extensive data augmentation in 3D was applied to account for overfitting. At test time, the algorithm was applied to overlapping patches over the entire image and resulting predictions were consolidated. Detection rates (= sensitivity) and FP rates per case were calculated. Table 2 provides information on important algorithm parameters. Supplement C provides further technical details.

**Table 2:** Important algorithm parameters

Parameter	Value
Data set split	60% train, 20% validation, 20% testing
Batch size	8*
Patch size	(192, 192, 32)*
Data augmentations	X-Y-rotations (360 degree), re-scaling (0.8–1.1), elastic deformations (alpha: 0,1500; sigma: 30,50))
Optimizer	Adam
Number of epochs	100
Initial number of feature maps	18
Preprocessing	(Clipping and scaling to [−1200, 600] followed by *z*-score normalization

*The optimal configuration of patch size versus batch size is a diligent task given the hardware constraints of limited GPU memory. Various combinations of the two parameters were tested on the validation set and the best model selected (batch size: 8, patch Size: 192, 192, 32)

### TNM categorization

In a subsequent step, all test cases were processed with a publicly available deep-learning lung segmentation algorithm with excellent performance (DICE Score: 0.98 ± 0.03) [17] and the following five anatomical regions were defined: (1) *lung region* (= segmentation mask resulting from the algorithm), (2) *mediastinum* (area between the two lung masks), (3) *bone* (HU-thresholding > 120 HU and filling holes), (4) *abdomen* (area beneath masks 1 and 2), and (5) *other* (all areas of the scan not covered by masks 1–4). Then, all lesions were attributed to the lesion subtypes as follows: T (all lesions within region 1), N (all lesions within region (2)), M (all lesions in region 3), and “N or M” (all lesions in region 4 or 5). Attribution accuracy was evaluated on the internal and external test set with original annotation labels as ground-truth.

### Analysis of reasons of FP and FN findings

All FP predictions of the Retina U-Net were visually checked and attributed to categories. FPs were assessed at the lowest classifier threshold possible (≥ 0.1) and at the threshold used for sensitivity analysis, which was defined by the threshold at which the FP/c rate drops below 2. We consider this to be a FP rate per case that is acceptable in a clinical environment. We conservatively rated repeated annotations of TP findings as FP. For example, if a lung tumor was detected three times, this was rated as 1 TP and 2 FP. The category (T, N, and M) of FNs was determined using the original lesion labels as ground truth.

### External validation

In addition to the internal test set, a total of 20 randomly selected data sets from patients with histologically proven primary lung cancer that underwent FDG-PET/CT (Biograph40, Siemens Healthineers; technical details in Supplement A) between 06/2021 and 09/2021 in another university hospital were processed with the algorithm after de-identification. Before processing, 3D ground-truth bounding boxes for all malignant lesions were drawn by R1 and R2 in consensus. Sensitivity and FPs per case were calculated and compared to the results based on the internal test set.

### Statistical analysis

Statistical analysis was performed using IBM SPSS Statistics for Windows, Version 22.0 (IBM Corp.). Scatter plots and graphs were created with scikit-learn [18]. Continuous data was described by means and standard deviation. Association between two or more categorical variables was tested with chi-square test. To test for statistical differences of means of continuous data of two or more groups, ANOVA or non-parametric alternatives were used depending on data structure. Normal distribution was assessed with histograms and Q-Q plots. *p* values less than 0.05 were defined to indicate statistical significance.

## Results

### Patient and tumor characteristics

Table 3 provides details on patient characteristics and tumor histology. Age and sex did not differ statistically significantly between training, validation, and internal test set (*p* = 0.32 and *p* = 0.50, respectively). There were no significant differences between the three internal data sets regarding tumor histology (*p* = 0.72). Patients from the internal and external test set were of comparable age  (*p* = 0.61), but more female patients were included in the external test set (*p* < 0.01) and the tumor histology mix was different (*p* < 0.01).

**Table 3:** Patient characteristics and tumor histology

	Internal data sets				
	Training set	Validation set	Testing set	Total	External testing set
Number of cases	216	74	74	364	20
Patient characteristics					
Age (mean/SD)	67.6 (10.2)	65.6 (10.2)	66.9 (10.8)	67.1 (10.3)	68.3 (8.5)
Sex (F/M; F%)	55/161 (25.5%)	24/50 (32.4%)	21/53 (28.4%)	100/264 (26.1%)	12/8 (60.0%)
Tumor histology					
Adenocarcinoma	114	41	40	195	6
Squamous cell carcinoma	56	21	22	99	0
NSCLC-NOS	18	5	2	25	2
Other	28	7	10	45	4
Unknown	0	0	0	0	8

The internal data sets comprised 364 PET/CTs with 576 T lesions (T1: *n* = 323; T2: *n* = 137; T3: *n* = 52; T4: *n* = 64) with a mean tumor volume of 39.9 cm^3^ (SD: 100.7 cm^3^), 1025 N lesions with a mean volume of 4.8 cm^3^ (SD: 12.4 cm^3^), and 312 M lesions with a mean volume of 6.2 cm^3^ (SD: 15.0 cm^3^). Mean volumes of malignant lesions were not statistically significantly different between the internal data sets (*p* = 0.09–0.65). The internal test set comprised 74 PET/CTs with 87 T lesions, 216 N lesions, and 76 M lesions. The external test set 20 PET/CTs with 64 T lesions, 76 N lesions, and 3 M lesions. The interreader IoUs were 0.86 (T lesions), 0.77 (N lesions), and 0.78 (M lesions).

### Internal performance analysis

In this section, all results are based on the internal test set.

#### General performance

**Fig. 4:**
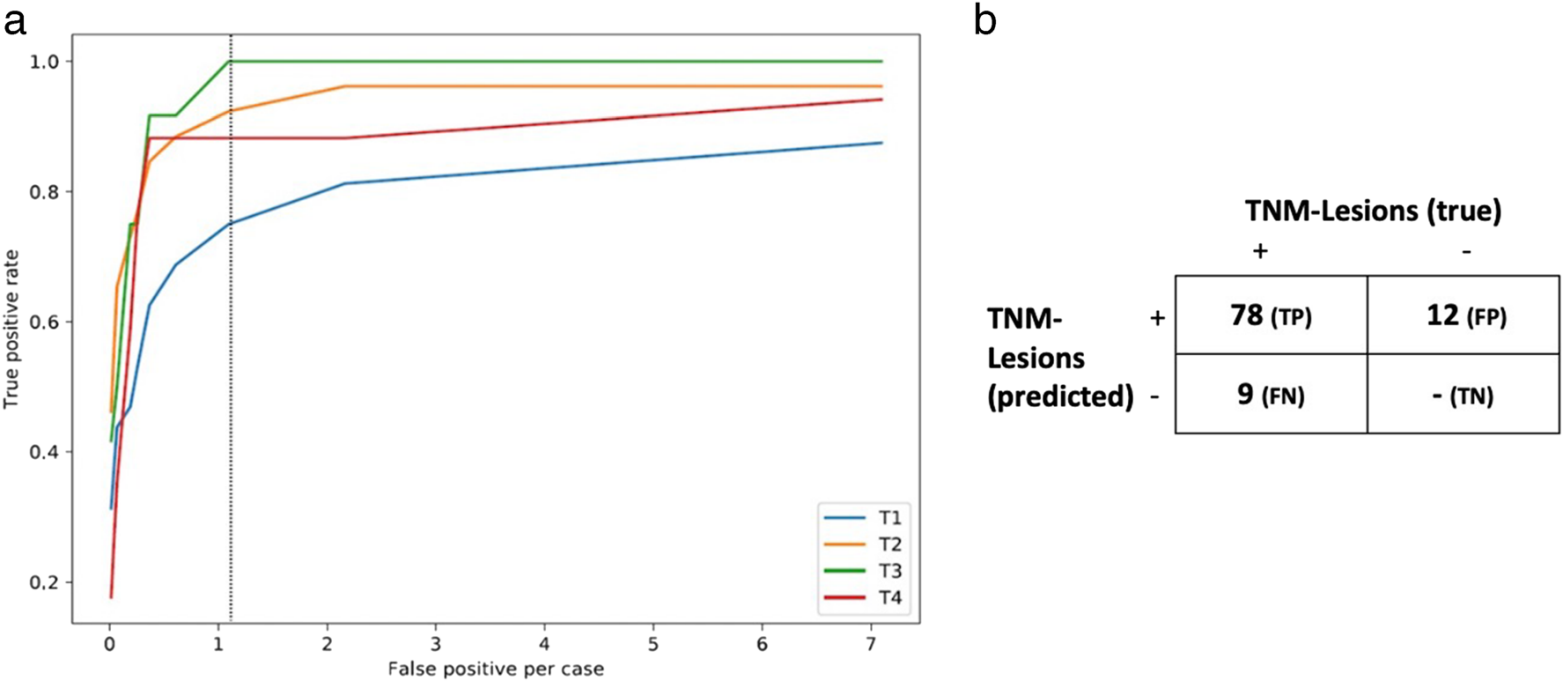
**a** FROC (free response ROC) curves for the T lesion detection task. Detection rate is higher for tumors of higher stage (T2–T4). The corresponding FROC scores, calculated as average TPR over FP/cs 0.125, 0.25, 0.5, 1, 2, 4, and 8 were 0.70 (T1), 0.88 (T2), 0.90 (T3), and 0.81 (T4). **b** A corresponding partial confusion matrix for the T lesion detection task. Please note that this is not a standard classification task, but an object detection task (“object versus background”), where by definition true negatives (TNs) are not defined (there are no “background objects”). Therefore, a number for TN is lacking by definition

**Fig. 5:**
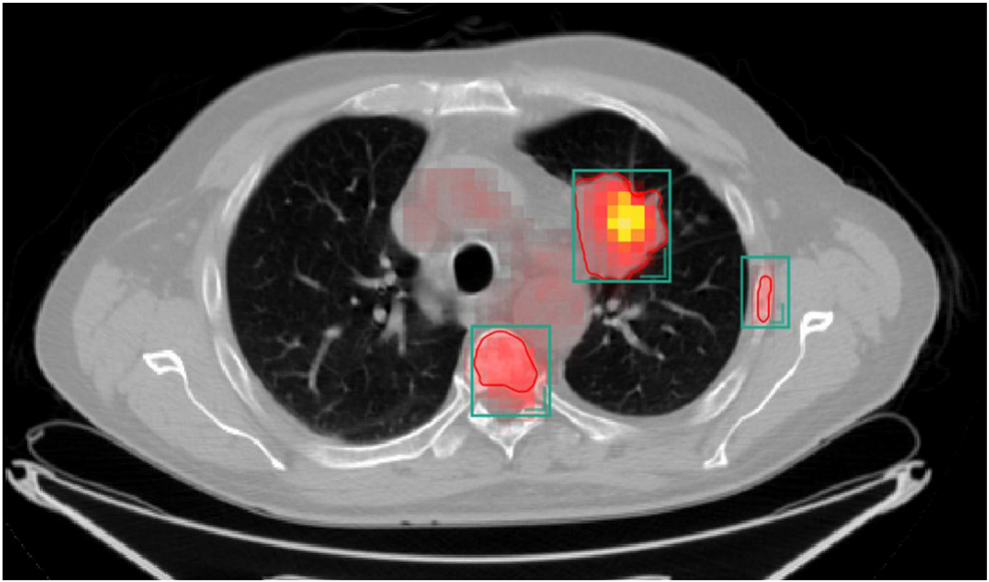
PET/CT of a 73-year-old male patient with an adenocarcinoma in the left upper lobe (T3) and two bone metastases (vertebra, left lateral rib) in axial orientation. The green bounding boxes are 3D lesion predictions, the output of the TNM Retina U-Net. The red lines delineate the 3D ground-truth segmentation masks. Based on this, a 3D ground-truth bounding box was created and compared with the lesion predictions via IoU

For reasons of clarity, detection rates (= sensitivities) are reported at a classifier threshold of 0.3 (equivalent to FP/c of 2.0). All sensitivities at all classifier thresholds for all T/N/M subcategories are provided in Supplement D. Overall sensitivity for T lesions was 86.2% (95% CI: 77.2–92.7). The T subcategory analysis revealed that sensitivity for more advanced lesions (T2/T3/T4) was higher compared to smaller T1 lesions: while all T3 lesions were detected (100% (95% CI: 73.5–100.0)), T1 tumors were more likely to be missed (detection rate 75.0% (95% CI: 56.6–88.5)). Sensitivity for N lesions wasere 54.2% (95% CI: 47.3–60.9) and metastases (M) were detected in 72.4% of cases (95% CI: 60.9–82.0). Figure 4 shows the FROC curves for the T detection task. Figure 5 shows an exemplary PET/CT of a patient with bone metastases and algorithm outputs as overlays. Finally, ESM 1 shows a video of detected lesions indicated as overlays on an original data set.

#### Analysis of false-positive findings

**Fig. 6:**
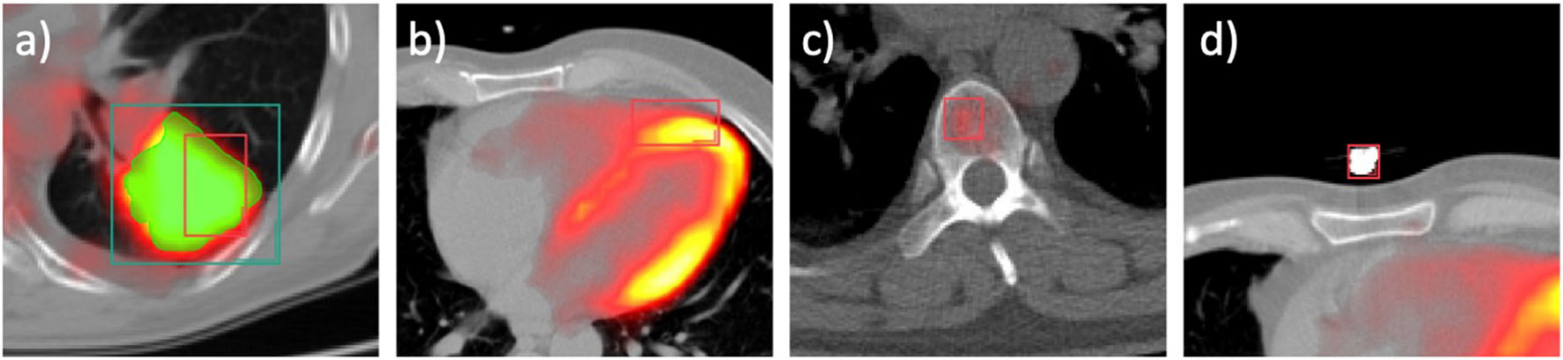
Four examples of FP findings (marked on PET/CT with red bounding boxes) due to (**a**) a double annotation of a true lung tumor that had already been detected (green bounding box), (**b**) the physiological activity of the myocardium, (**c**) the physiological activity of bone marrow, and (**d**) an extracorporal foreign body

At the lowest classifier threshold (0.1), the model produced 1227 FPs. Most were caused by double annotations of a true malignant lesion (*n* = 288, 23.5%), followed by the physiological activity of bone marrow with 16.8% (*n* = 206), mediastinal structures with 13.4% (*n* = 164; of which *n* = 48 were caused by the physiological activity of the myocardium), and pulmonary structures with 13.0% (*n* = 159; e.g., consolidations, lung vessels). Of note, only slightly increasing the classifier threshold to 0.3 reduced the number of FPs by 87.8%, to 150. At this threshold, main causes for FP were double annotations of true malignant lesions (*n* = 68, 45.3%), abdominal structures (*n* = 25, 16.7%), and mediastinal structures (*n* = 18, 12%). Table 4 provides a comprehensive analysis of reasons of FPs. Figure 6 shows four examples of FP findings.

**Table 4:** Detailed account on structures causing FP findings in the internal testing set at the lowest possible classifier threshold of 0.1 and a threshold of 0.3. A significant drop in FPs is observed by increasing the classifier threshold

Cause	Classifier threshold 0.1		Classifier threshold 0.3	
	*n*	%	*n*	%
(1) Double annotation of TP	288	23.5	68	45.3
(2) Bones	206	16.8	16	10.7
Ribs	107		7	
Vertebrae	60		6	
Other bones	39		3	
(3) Mediastinal structures	164	13.4	18	12.0
Heart	48		7	
Mediastinal vessels	30		3	
Esophagus	27		2	
Other structures	59		6	
(4) Pulmonary structures	159	13.0	12	8.0
Pulmonary vessels	97		2	
Consolidation/ground-glass opacity	50		2	
Other	12		8	
(5) Abdominal structures	126	10.3	25	16.7
Kidney	39		12	
Gastrointestinal structures	35		7	
Liver	26		1	
Other structures	26		5	
(6) Tubes and lines (foreign bodies)	39	3.2	0	0
(7) Other	245	20.0	11	7.3
Soft tissue (non-muscle)	97		3	
Muscle	69		5	
Other	79		3	
Total	1227	100	150	100

#### Analysis of false-negative findings

Of 76 missed findings, 60 were lymph nodes (78.9%), 10 metastases (13.2%), and 6 tumors (7.9%; T1: *n* = 4; T2: *n* = 1; T3: *n* = 0; T4: *n* = 1).

#### TNM classification with anatomical region definitions

Of 247 detected lesions, based on their location within the automatically defined anatomical regions, 233 (94.3%) were correctly classified as T, N, or M. Of the wrong classifications, 85.7% (*n* = 12/14) were caused by T lesions being misclassified as N or M, mostly in the area of the hili as contact zone of lung mask and mediastinal mask. Supplement E shows an example image with anatomical region masks.

### External validation

Overall sensitivity for the detection of malignant lesions was 88.8% (95% CI: 82.5–93.5%, *n* = 127/143; at an FP/c of 2.7). Detection rates for T lesions were 93.8% (95% CI: 84.8–98.3%, *n* = 60/64), for N lesions 84.6% (95% CI: 74.0–91.6%, 64/76), and for M lesions 100% (95% CI: 29.2–100.0%, *n* = 3/3). Based on the anatomical regions, 110 of 129 detected lesions (= 85.3%) were correctly classified as T, N, or M. Of the wrong classifications, most (*n* = 15/17; 88.2%) were caused by T lesions being misclassified as N or M.

## Discussion

Our analysis of a Retina U-Net algorithm revealed good overall detection rates of malignant lesions on PET/CT at low false-positive rates per case. In this scenario, the Retina U-Net integrates the information from both CT (anatomical) and PET (metabolic) components. Slightly lower detection performance for T1 tumors was noticed, compared to larger T2–T4 lesions. This is an interesting finding and is most probably explained by the fact that small tumors are affected by partial volume effect, which is especially relevant in PET with lower spatial resolution compared to high-resolution lung CT. Furthermore, the CT component of FDG-PET/CT is routinely acquired in free breathing and motion artifacts exhibit more influence on the detection of small lesions compared to advanced tumors.

Regarding automated lesion detection on PET/CT, Teramoto et al evaluated a method for detection of pulmonary nodules (average diameter: 19 mm) in 104 patients [12]. The nodules were detected on CT and PET images separately, based on active contour filtering and thresholding, respectively. After using a convolutional neural network (CNN) for FP-reduction, the FP/c was 4.9 and the detection rate was 90.1%. In opposition to this multi-step, mainly hard-coded processing pipeline, the Retina U-Net presented in this article is an end-to-end approach, which has the advantage of potential optimization by adding more training data. Schwyzer et al trained a model to perform a binary classification whether there is a nodule present in a single CT image or not [13]. This approach has strong limitations such as discarding all 3D information by only operating on 2D slices and incapability of handling multiple lesions per slice. Furthermore, training with whole slice binary labels only as opposed to annotations of individual nodules and their associated locations is known to be data inefficient and prone to overfitting, since the algorithm requires large amounts of data in order to relate the binary label to certain image regions without supervision. Finally, Kirienko et al used a CNN to classify T1–T2 vs. T3–T4 stages in 472 patients (T1–T2: *n* = 353, T3–T4: *n* = 119) using 3D bounding boxes, cropped around the center of the lesions on both PET and CT images [19]. They reached an accuracy of 69.1%, sensitivity of 70.0%, and specificity of 66.7%. Our approach is more granular and also encompasses N and M lesions. Furthermore, it resulted in higher sensitivity on both internal and external test data. An interrater variability analysis showed slightly higher levels of agreement compared to Borrelli et al [20].

Our approach yielded highest detection rates for T lesions. Detection rates of N and M lesions were lower, probably explained by the anatomical surroundings that provide a stark contrast for T lesions (high attenuation [tumor] vs. low attenuation [lung parenchyma]), but not so much for N and M lesions that are surrounded by tissues of similar density. All false positive detections had an anatomical correlate, such as the physiological metabolism of the myocardium. The fact that no arbitrary lesions were detected reinforces our trust in the Retina U-Net algorithm and seems to be in line with radiological judgment and decision-making. Of note, the FP/c at the chosen threshold dropped from 2.0 to 1.1 when removing those FPs that were caused by double annotations of true lesions—an approach that seems fair keeping in mind that it is technically easily implementable.

External validation yielded better results than internal testing. This is unusual, but can be explained by the different structure of the external data set regarding tumor histology and lesion type mix: its T vs. N/M ratio was higher compared to the internal test set. Other factors influencing the performance on the external data set are differences in hardware (PET/CT scanner) and, given the small number of examinations in the external data set, coincidence. While the internal data set comprised 364 examinations and is among the largest used in the context of AI and PET/CT, the task of lesion sub-categorization drastically decreases the amount of training cases per category. Thus, for this initial study, we opted for the simpler task of general lesion detection, i.e., grouping lesions into one foreground class to be distinguished from background, and perform detailed TNM-classification with a second approach (anatomical regions). We are aware that this has the disadvantage of introducing an extra layer of uncertainty that decreases general performance of the whole processing pipeline, although the anatomical regions approach correctly attributed 94.3% of detected lesions.

In the future, the algorithm could serve physicians by reducing false-negative calls, reducing the time needed to analyze staging PET/CTs, and allowing for advanced evaluations such as automated tumor volume quantification.

Our study has limitations. First, ground-truth data originated from one center and lesions were manually segmented by two readers in random order without double reading, but with supervision. However, literature reports high ICCs between human readers for tumor delineation in PET/CT with ICCs ranging between 0.987 and 0.995 [21], which was confirmed in this study by high IoU values on a subset of 60 lesions. Second, volume cropping to the chest was applied and therefore extra-thoracic lesions were not completely considered. This has the advantage of avoiding FPs caused by non-tumorous organs with physiologically high PET signal like the urinary bladder or the brain. At the same time, the algorithm has not been tested for detection of metastases (N, M) in the whole body. Third, even though the data set was large, the 95% CIs were wide. This is because the internal test set encompasses only 20% of the whole internal data set. Fourth, the performance, especially regarding N and M lesions, is currently not good enough for a stand-alone application and algorithm results warrant validation by a physician trained in nuclear medicine.

In conclusion, the Retina U-Net algorithm is well suited for the 3D detection of lung cancer lesions in PET/CT. To further advance the methodology towards clinical application, the approach will be expanded to the whole body. To this end, besides a swift and intuitive workflow allowing for modification of automatically generated results, more extrathoracic annotations are needed.

### Accessibility

The framework and its full configuration used in this study is available on Github: https://github.com/MIC-DKFZ/medicaldetectiontoolkit/tree/master/experiments/pet_ct_tnm_classification. 3D Slicer and associated plugins are available at https://github.com/Slicer/slicer.

## Supplementary Information

ESM 1 (DOCX 802 kb)

ESM 2 (MP4 2109 kb)

## Abbreviations

AI: Artificial intelligence
CNN: Convolutional neural network
FN: False negative
FP: False positive
FP/c: False positives per case
FROC: Free response receiver operating characteristic curve
GPU: Graphics processing unit
IoU: Intersection-over-union
M: Metastasis (distant metastasis)
N: Node (lymph nodes)
T: Tumor (main tumor)
TN: True negative
TP: True positive

## Declaration

### Guarantor

The scientific guarantor of this publication is Prof. Alexander W. Sauter.

### Statistics and biometry

No complex statistical methods were necessary for this paper.

### Methodology

• retrospective

• experimental

• multicenter study
