# Supplementary material for: Automated lung cancer assessment on 18F-PET/CT using Retina U-Net and anatomical region segmentation
Source: Eur Radiol. 2023 Jan 10;33(6):4270–9. doi: 10.1007/s00330-022-09332-y (PMC10182147; doi:10.1007/s00330-022-09332-y)
Supplement: Supplementary file 1 — (DOCX 802 kb) [file 330_2022_9332_MOESM1_ESM.docx]

**Supplement A.** Technical details on the PET/CT examinations

Internal dataset

The CT component of the combined PET/CT examination was acquired with the following parameters. Discovery STE (GE Healthcare): slice thickness 3 mm, i50f kernel, x-ray tube voltage 120 kVp (SD: 0 kVp), tube current 79.4 mAs (SD: 15.0 mAs), CTDIvol 5.8 mGy (SD: 1.7 mGy) and dose length product (DLP) 536.6 mGy*cm (SD: 99.1 mGy*cm). Biograph mCT-X (Siemens Healthineers): slice thickness 3 mm, i50f kernel, x-ray tube voltage 120 kVp (SD: 0 kVp), exposure 37.2 mAs (SD: 17.2 mAs), CTDIvol 3.1 mGy (SD: 1.4 mGy) and DLP 294.0 mGy*cm (SD: 144.3 mGy*cm). In 21 cases, a contrast agent at mean dose of 87.1 ml (SD: 24.9 ml) was used (Iopromide, Ultravist 370, Bayer Pharma). The rest of the examinations were performed without contrast. Attenuation-corrected PET images of the Discovery STE (GE Healthcare) were reconstructed using the standard proprietary 3-dimensional iterative reconstruction (3 mm in full width at half maximum, 21 subsets, 2 iterations, and 128 × 128 matrix). For the Biograph mCT-X (Siemens Healthineers) a reconstruction with OSEM3D+TOF with 21 subsets, 2 iterations, 128 × 128 matrix and 5 mm Gauss convolutions kernel was applied.

External dataset

Scans were obtained on a Biograph 40 (Siemens Healthineers) and one hour after intravenous injection of 5 MBq FDG/kg body weight at glycemic levels below 10 mmol/L and previous fasting for at least 6 hours. Slice thickness was 3 mm, X-ray tube voltage 120 kVp (SD: 0 kVp), exposure 155 mAs (SD: 38.8 mAs), CTDIvol 10.9 mGy (SD: 3.5 mGy) and dose length product (DLP) 1119.1 mGy*cm (SD: 296.5 mGy*cm). Attenuation-corrected PET images were reconstructed using 3D OP-OSEM PSF+TOF (point spread function and time of flight correction) reconstruction with 21 subsets, 3 iterations, and a 200 x 200 matrix (FWMH: 2 mm).

**Supplement B.** Exploratory analysis of three different models

An exploratory analysis of three different models was performed: the T-model was trained with T-lesions only, the TN-model using T & N-lesions, the TNM-model with T, N & M-lesions. The TNM-model performed best compared to the other models (average precision [AP] for the T-task: 0.71; TN-model: 0.65; T-model: 0.63). This was expected, as more examples were used for training of the TNM-model. As a consequence, and because the aim was a comprehensive approach predicting all malignant lesions, the TNM approach was further developed and analyzed in-depth.

**Supplement C.** Technical details on the Retina U-Net

Training was performed on patch crops of size (192, 192, 32). To account for the class-imbalance of object level classification during loss computation, the hardest negative object candidates according to softmax probability were stochastically mined. Models were trained with a batch size of 8 using the Adam optimizer (with default settings). The learning rate was initialized at 10e−4 and set to 5*10e-5 after half of the training and to 10e-5 after 75% of the training time. Extensive data augmentation in 3D is applied to account for overfitting using the batchgenerators package]:  XY-rotations, elastic transformations, and re-scaling of images with facots (0.8-1.1) [details: see reference below]. At test time, the classifier threshold was set to 0.3, both during internal and external testing. The full configuration is available on GitHub: https://github.com/MIC-DKFZ/medicaldetectiontoolkit/tree/master/experiments/pet_ct_tnm_classification.

---

Reference: Isensee, F., Jäger, P., Wasserthal, J., Zimmerer, D., Petersen, J., & Kohl, S. (2020). batchgenerators—a python framework for data augmentation. 2020.

**Supplement D.** Detailed data on sensitivities according to TNM subcategories at various classifier thresholds for the TNM-approach

|  | **Classifier Threshold** | **Lesion subtype** | **Sensitivity % (Lesions detected / All lesions)** |  | **Classifier Threshold** | **Lesion subtype** | **Sensitivity % (Lesions detected / All lesions)** |  | **Classifier Threshold** | **Lesion subtype** | **Sensitivity % (Lesions detected / All lesions)** |
| --- | --- | --- | --- | --- | --- | --- | --- | --- | --- | --- | --- |
| **TNM** | **0.1** | T1 | 87.5% (28/32) |  | **0.2** | T1 | 81.3% (26/32) |  | **0.3** | T1 | 75.0% (24/32) |
|  | FP/c=16.6 | T2 | 96.2% (25/26) |  | FP/c=5.0 | T2 | 96.2% (25/26) |  | FP/c=2.0 | T2 | 92.3% (24/26) |
|  |  | T3 | 100.0% (12/12) |  |  | T3 | 100.0% (12/12) |  |  | T3 | 100.0% (12/12) |
|  |  | T4 | 94.1% (16/17) |  |  | T4 | 88.2% (15/17) |  |  | T4 | 88.2% (15/17) |
|  |  | N1 | 60.0% (27/45) |  |  | N1 | 55.6% (25/45) |  |  | N1 | 44.4% (20/45) |
|  |  | N2 | 73.7% (73/99) |  |  | N2 | 67.7% (67/99) |  |  | N2 | 54.5% (54/99) |
|  |  | N3 | 77.8% (56/72) |  |  | N3 | 70.8% (51/72) |  |  | N3 | 59.7% (43/72) |
|  |  | M | 86.8% (66/76) |  |  | M | 76.3% (58/76) |  |  | M | 72.4% (55/76) |
|  |  |  |  |  |  |  |  |  |  |  |  |
|  | **0.4** | T1 | 68.8% (22/32) |  | **0.5** | T1 | 62.5% (20/32) |  | **0.6** | T1 | 53.1% (17/32) |
|  | FP/c=1.4 | T2 | 88.5% (23/26) |  | FP/c=0.8 | T2 | 84.6% (22/26) |  | FP/c=0.5 | T2 | 76.9% (20/26) |
|  |  | T3 | 91.7% (11/12) |  |  | T3 | 91.7% (11/12) |  |  | T3 | 75.0% (9/12) |
|  |  | T4 | 88.2% (15/17) |  |  | T4 | 88.2% (15/17) |  |  | T4 | 76.5% (13/17) |
|  |  | N1 | 31.1% (14/45) |  |  | N1 | 28.9% (13/45) |  |  | N1 | 26.7% (12/45) |
|  |  | N2 | 51.5% (51/99) |  |  | N2 | 41.4% (41/99) |  |  | N2 | 30.3% (30/99) |
|  |  | N3 | 47.2% (34/72) |  |  | N3 | 34.7% (25/72) |  |  | N3 | 31.9% (23/72) |
|  |  | M | 60.5% (46/76) |  |  | M | 53.9% (41/76) |  |  | M | 43.4% (33/76) |
|  |  |  |  |  |  |  |  |  |  |  |  |
|  | **0.7** | T1 | 46.9% (15/32) |  | **0.8** | T1 | 43.8% (14/32) |  | **0.9** | T1 | 31.3% (10/32) |
|  | FP/c=0.3 | T2 | 73.1% (19/26) |  | FP/c=0.1 | T2 | 65.4% (17/26) |  | FP/c=0.03 | T2 | 46.2% (12/26) |
|  |  | T3 | 75.0% (9/12) |  |  | T3 | 50.0% (6/12) |  |  | T3 | 41.7% (5/12) |
|  |  | T4 | 58.8% (10/17) |  |  | T4 | 35.3% (6/17) |  |  | T4 | 17.6% (3/17) |
|  |  | N1 | 22.2% (10/45) |  |  | N1 | 13.3% (6/45) |  |  | N1 | 4.4% (2/45) |
|  |  | N2 | 22.2% (22/99) |  |  | N2 | 16.2% (16/99) |  |  | N2 | 7.1% (7/99) |
|  |  | N3 | 25.0% (18/72) |  |  | N3 | 13.9% (10/72) |  |  | N3 | 2.8% (2/72) |
|  |  | M | 27.6% (21/76) |  |  | M | 18.4% (14/76) |  |  | M | 7.9% (6/76) |
|  |  |  |  |  |  |  |  |  |  |  |  |

**Supplement E.** PET/CT of a patient with adenocarcinoma (T2) in the right upper lobe in axial (a), coronal (b), and sagittal (c) view. The green boxes indicate the 3D lesion prediction bounding box. Colors indicate the anatomical regions bone (yellow), mediastinum (blue), and abdomen (purple). The lung region is not color-labelled for better visibility.


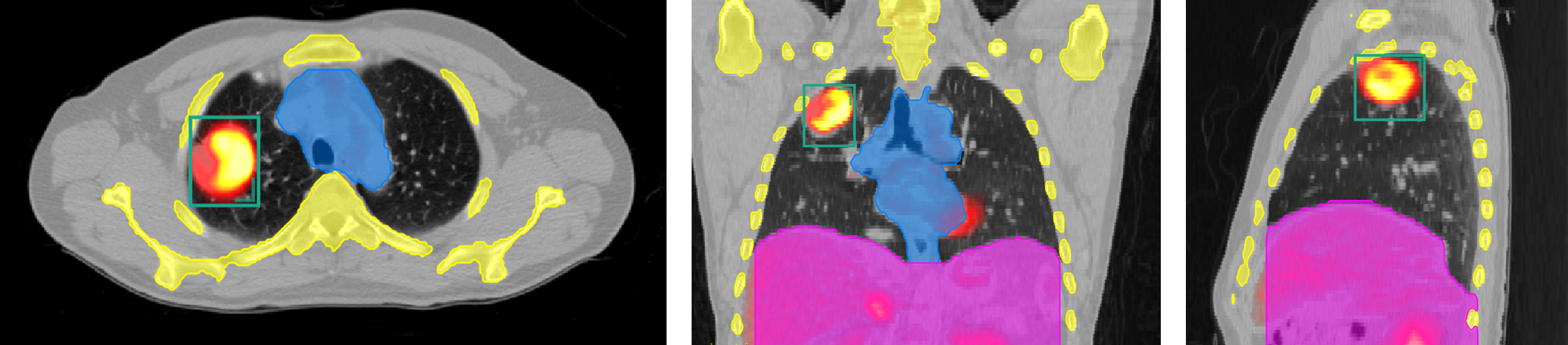


**Electronic Supplementary Material (ESM) 1**

This video shows the algorithms’ output (predictions of malignant lesions) as green bounding boxes on the data input, a PET/CT of a patient with lung cancer and associated metastases (duration: 16 seconds).
